# Enhanced H_2_ sorption performance of magnesium hydride with hard-carbon-sphere-wrapped nickel

**DOI:** 10.1039/c8ra05464a

**Published:** 2018-08-13

**Authors:** Dandan Peng, Zhenmin Ding, Yaokun Fu, Yu Wang, Jia Bi, Yuan Li, Shumin Han

**Affiliations:** State Key Laboratory of Metastable Materials Science and Technology, Yanshan University Qinhuangdao 066004 P. R. China; School of Environmental and Chemical Engineering, Yanshan University Qinhuangdao 066004 P. R. China hanshm@ysu.edu.cn +86-335-8074648 +86-335-8074648

## Abstract

Magnesium hydride is regarded as one of the most ideal candidates for hydrogen storage, but its relatively high operating temperatures and slow kinetics always hinder its commercial applications. Herein, we first fabricated hard-carbon-sphere-wrapped Ni (Ni/HCS) *via* a mild chemical method; subsequently, the as-prepared additive was introduced to fabricate an Mg–Ni/HCS composite by using hydriding combustion synthesis. Hard carbon spheres (HCS) effectively inhibited the agglomeration of hydride particles during hydrogen storage cycling; they could also provide active sites to promote the nucleation of Mg-based hydrides. During the hydriding combustion synthesis procedure, *in situ*-formed Mg_2_NiH_4_ could induce the absorption of MgH_2_, thus triggering its hydrogen properties. Remarkable enhancement in hydrogen absorption properties of the composite was found. The composite absorbed 6.0 wt% H_2_ within 5 min at 275 °C; moreover, even at 75 °C, it could still absorb 3.5 wt% H_2_. Furthermore, it delivered a high reversible hydrogen absorption capacity of 6.2 wt% and excellent rate capability at 350 °C. It was also demonstrated that the composite could release 6.2 wt% H_2_ at 350 °C within 5 min. A rather low activation energy value (65.9 kJ mol^−1^) for the dehydrogenation of MgH_2_ was calculated as compared to that for commercial MgH_2_ (133.5 kJ mol^−1^).

## Abstract 2

Magnesium hydride is regarded as one of the most ideal candidates for hydrogen storage, but its relatively high operating temperatures and slow kinetics always hinder its commercial applications.

## Graphical abstract

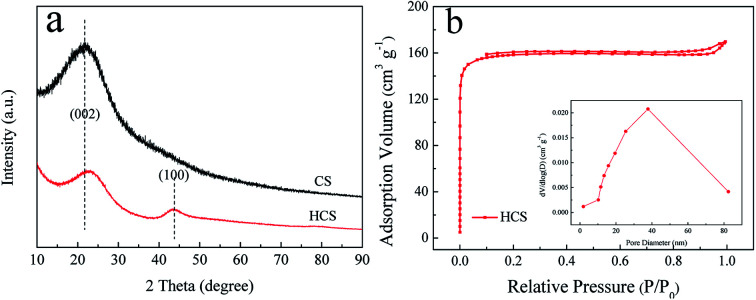

## 1. Introduction

One of the most technically challenging issues anticipated for future hydrogen economy is the development of safe and efficient onboard hydrogen storage materials.^1^ A variety of materials that satisfy the requirements of sufficient storage density, beneficial thermodynamics, desirable kinetics, and low cost have been studied in pursuit of a suitable hydrogen storage system.^2,3^ Among solid-state hydrogen storage systems, magnesium-based hydrides have been proposed to be promising candidates for effective hydrogen storage due to their high volumetric and gravimetric hydrogen storage capacities, high abundance, low cost, nontoxicity, and high safety.^4^ However, their unfavorable thermodynamic stability and slow kinetic behavior significantly obstruct their commercial use, and proper strategies are required to overcome these problems. During the past decade, many methods have been discovered and proven to be effective for magnesium hydride systems, and they include both chemical and physical methods.^5–11^ One of these methods involves alloying with transition metal elements (*e.g.*, Mg_2_Ni); Mg-based alloys exhibit better thermal instability of hydrides than pure magnesium.^12–15^ However, the unavoidable loss of hydrogen storage capacity is the major drawback of the alloying route due to the addition of a transition metal. To solve this problem, a beneficial solution is to build MgH_2_-rich complex hydrides,^16^*i.e.*, MgH_2_–Mg_2_NiH_4_ composites, which exhibit a considerable reduction in stability without a clear loss in capacity.^14,17^ Recently, the introduction of various novel catalysts has been used as another strategy to relieve the kinetic barrier and thermodynamic stability of magnesium hydride systems.^18–24^ Catalysts are usually supposed to function as activation agents, forming intermediate metastable states, facilitating dynamics, and destabilizing the Mg–H bonding energy. Furthermore, another very effective technique to relieve the kinetic barrier and thermodynamic stability of Mg-based hydrides is nanostructuring,^25^ which can directly result in larger surface-to-volume ratio of the particles and shorter solid-state diffusion distances for hydrogen. Recently, extensive experimental and theoretical studies have demonstrated that decreasing the particle size is an efficient way to destabilize Mg-based hydrides, leading to further enhancement in hydrogen storage performances.^26–30^ However, high reactivity of Mg-based hydrides restricts the synthetic methods for nanostructuring because of severe agglomeration. Meanwhile, owing to high surface energy, the original hydride nanoparticles always tend to aggregate based on the solid-state reactions, leading to rapid loss of the initial morphology and continual reduction in storage capacities.^31^ Previously, Imamura and coworkers had reported studies on catalyzing MgH_2_ with graphite;^32,33^ since then, many carbon materials, including multiwalled carbon nanotubes (MWCNT),^34^ single-walled carbon nanotubes (SWCNT),^35–37^ graphene nanosheets (GNS),^38^ and activated carbon,^39^ have been extensively studied to promote the hydrogen storage properties of MgH_2_. Although these issues can be solved by dispersing MgH_2_ into porous carbon hosts, these strategies unfortunately suffer from irregular spatial distributions and low infiltration efficiency.^40,41^

As the demand for renewable energy continues to grow, efforts toward improving the efficiency of carbon materials are getting more and more attention. Yuan *et al.*^42^ studied Mg-based hydrides associated with carbon-based catalysts; by focusing on the roles of metal-assisted MWCNT hybrids, they revealed that MWCNTs and supporting metallic particles have a synergetic catalytic effect. Meanwhile, Yao *et al.*^43^ also reported the synergistic effect of metallic couple and carbon nanotubes on Mg, yielding ultrafast kinetics due to metal–H atomic interactions at the Mg surface. In addition, Ismail and coworkers^44^ showed that 10 wt% K_2_NbF_7_ and 5 wt% MWCNT milled with MgH_2_ significantly reduced the initial decomposition temperature to 248 °C with a capacity of 6.2 wt%; they also found that co-doping FeCl_3_ and CNTs with MgH_2_ resulted in both decreased desorption temperature and improved sorption kinetics when compared to those of the undoped MgH_2_–FeCl_3_ composite.^45^ Amirkhiz *et al.*^46^ examined the effect of SWCNT-metallic nanoparticle additions on the hydrogen desorption behavior of MgH_2_; after high-energy co-milling, they found that SWCNT-nanoparticle additions have beneficial influence on the desorption kinetics. Furthermore, in the carbon family, hard carbon spheres (HCS) have attracted tremendous attention due to their notable physical and chemical properties. For example, Yang *et al.*^47^ studied monodispersed hard carbon spherules as a catalyst support for the electrooxidation of methanol. Moreover, because of their superior surface area and controllable homogeneous structure, carbon spheres can provide several promising applications in energy storage. Therefore, the introduction of HCS as a support in Mg-based hydride systems has considerable potential for significantly enhancing the de/absorption properties.

Herein, we controllably fabricate hard carbon sphere-wrapped Ni additives by a chemical method and then, we introduce these materials into Mg-based hydride systems; significant enhancement in their de/absorption behaviors has been observed. Moreover, a possible mechanism for the catalytic phase in the system has been proposed.

## 2. Experimental

### 2.1 Synthesis of Ni/HCS and Mg–Ni/HCS

All chemicals were of analytical grade and used without any further purification. The typical preparation process of HCS was based on a solvothermal method followed by a calcination procedure at high temperatures. During the hydrothermal process, we selected sucrose (Aladdin, AR, 0.28∼0.90 mm) as the precursor; then, the precursor was subjected to a dewatering process, yielding carbon spheres. An aqueous sucrose solution of 0.2 mol L^−1^ was filled in a Teflon-lined autoclave at a fill rate of 80%, which was heated gradually to 190 °C and maintained at this temperature for 5 h. The obtained black precipitated particles were separated by filtration and washed with deionized water and ethanol (Aladdin, AR, ≧99.5%), and this process was repeated several times. The prepared black powders were fully dried in vacuum at 60 °C for 24 h to obtain a solid product, namely, carbon spheres (CS). Then, the as-prepared CS were further carbonized at 950 °C for 4 h under an argon atmosphere in a tube furnace. Then, the product was naturally cooled and transferred into a glovebox for further handling; the obtained final product was denoted as HCS. Then, HCS were initially treated with concentrated nitric acid (Aladdin, AR, 65–68%) to introduce carbonyl, carboxylic, and hydroxyl groups onto the surfaces; these functional groups may provide nucleation sites for metal species. To synthesize Ni/HCS, we mixed HCS and Ni(NO_3_)_2_·6H_2_O (Aladdin, AR, 98%) at a mass ratio of 6 : 4 (Ni : HCS) in 50 mL acetone (Sigma-Aladdin, AR, ≧99.5%); this mixture was subjected to ultrasonic treatment for half an hour and dried in a blowing dry oven at 50 °C for 8 h. Afterwards, the product was heat-treated in a tube furnace at 400 °C for 4 h under an Ar atmosphere. Finally, the products were reduced under an H_2_ atmosphere at 450 °C for 24 h.

The Mg–Ni/HCS composite was synthesized *via* hydriding combustion synthesis of Mg powder (Shanghai Pantian nano Materials Co., 99.9%, 1 μm), yielding Ni/HCS with a mass ratio of 5 : 1. In the hydriding combustion synthesis process, 0.75 g of crude magnesium powder and 0.15 g Ni/HCS were mixed in a pressure reactor vessel and heated to 400 °C under a hydrogen pressure of 4 MPa for 40 h. Subsequently, the sample was mechanically milled in a stainless steel vial with a ball-to-powder weight ratio of 30 : 1 under an Ar atmosphere. The ball milling process was paused for 15 min after each 15 min of milling to avoid increasing the temperature of the sample. For comparison, an Mg–HCS composite was synthesized under the same conditions, and pure MgH_2_ (Aldrich, 95%) was also prepared by a planetary ball mill under the same conditions. All the materials were handled in a glove box filled with purified argon (99.999%), which could maintain the water vapor and oxygen levels below 1 ppm by recycling the purification system to avoid oxidation.

### 2.2 Structural analysis and hydrogen desorption experiments

A Rigaku SmartLab X-ray diffractometer (XRD) with Cu Kα radiation was used to complete phase analysis. During the sample transfer and scanning process, an argon-filled container was used to prevent the samples from contacting air and moisture. The microstructure was further examined by transmission electron microscopy (TEM, JEM-2010, working at 120 kV) equipped with energy dispersive analysis by X-ray spectroscopy (EDAX). A scanning electron microscope (SEM, S-3400) was used to observe the surface morphology, and nitrogen flow was applied to prevent H_2_O/O_2_ contamination during the transfer process. The specific surface areas and porous nature of the as-prepared HCS were further, investigated by nitrogen adsorption/desorption measurements (ASAP 2020 Plus). Brunauer–Emmett–Teller (BET) and Barrett–Joyner–Halenda (BJH) methods were used to calculate the surface area and pore size distribution, respectively, based on the desorption data. The thermal behavior of the sample was investigated by thermal analysis (DTG-60A) from room temperature to 500 °C at different heating rates (5, 10, 15, 20 °C min^−1^). The valence states and structural information of the synthesized Ni/HCS were determined using an X-ray photoelectron spectrometer (XPS).

The hydrogen desorption and absorption properties of the as-synthesized Mg–Ni/HCS and Mg–HCS were determined using Sieverts-type pressure-composition-temperature (PCT). During isothermal rehydrogenation experiments, the composites were pressurized at 3 MPa. For the cyclic stability measurements of the composite, we performed dehydrogenation under 0.001 MPa for 2 h, and the hydrogenation process was carried out at 3 MPa for 1 h.

## 3. Results and discussion

### 3.1 Characterization of Mg–Ni/HCS and Mg–HCS

**Fig. 1:**
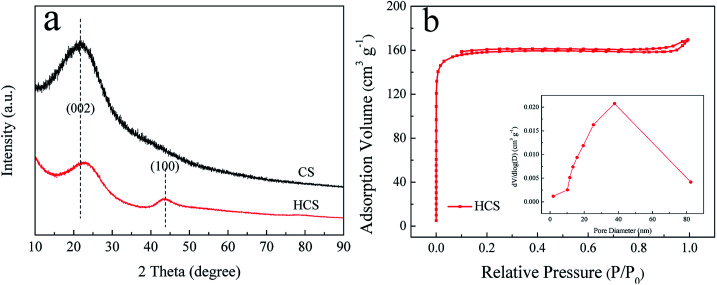
X-ray diffraction (XRD) profiles (a) of the obtained CS and HCS; N_2_ adsorption–desorption isotherms and the corresponding pore size distribution curves (b) of HCS.

Fig. 1a shows the XRD patterns of the as-synthesized CS and HCS. From the XRD patterns, it is evident that CS possess an amorphous structure when subjected to a hydrothermal process at 190 °C for 5 h, with just one broad reflection peak at around 2*θ* = 22° corresponding to the (002) plane. However, two broad peaks around 23° and 44° corresponding to the (002) and (100) reflections of the graphite layers are observed after carbonization in the tube furnace, indicating that the second process yields graphitization of HCS. Moreover, the peak of (002) shifts slightly to a higher 2*θ* value after the second carbonization process. The specific surface areas and features of HCS are further investigated by nitrogen adsorption/desorption measurements. BET analysis (Fig. 1b) indicates that the as-prepared HCS have a specific surface area and pore volume up to 623 m^2^ g^−1^ and 0.22 cm^3^ g^−1^, respectively. The pore size distributions are calculated from the desorption isotherms by the BJH method, and the peak in the BJH pore distribution is at 40 nm, exhibiting the characteristics of a mesoporous material. This is in good agreement with the following microscopy findings (Fig. 3d). The high surface area of HCS with mesoporous materials can undertake the role of a mechanical support, and it is believed that HCS can easily anchor and load Ni- and Mg-based composites with uniform dispersion; this indicates that it can play an important role in mediating the nucleation and growth of Mg-based particles coupled with this hydrogenation process strategy.

**Fig. 2:**
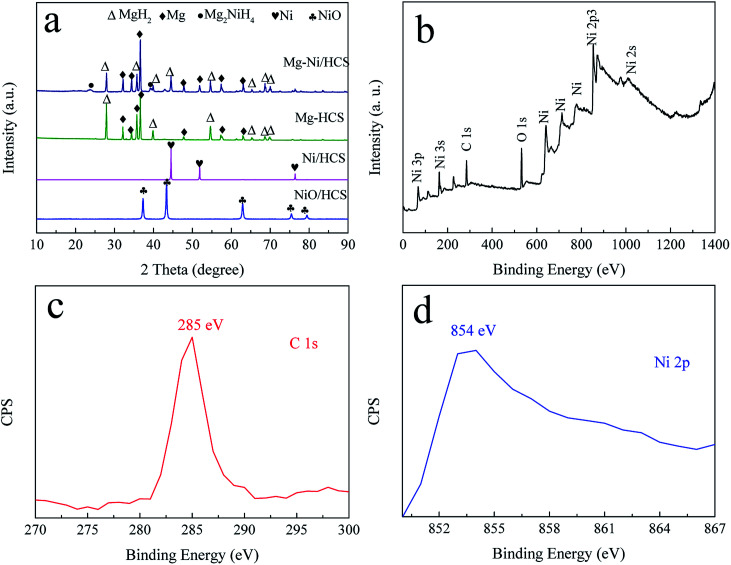
XRD curves (a) of the prepared Ni/HCS, Mg–HCS, and Mg–Ni/HCS, and XPS spectrum (b–d) of the as-synthesized Ni/HCS sample.

Fig. 2a shows the XRD patterns of Ni/HCS, Mg–Ni/HCS, and Mg–HCS composites before ball milling. As shown in Fig. 2a, the acetone solution of HCS and Ni(NO_3_)_2_·6H_2_O was dried and heat-treated in a tube furnace under an Ar atmosphere. Sharp diffraction peaks of NiO phases appeared as the characteristic peaks; after reducing in an H_2_ atmosphere, the NiO phase disappeared, and the peaks centered at 44.5° (111), 51.7° (200), and 76.4° (220) were assigned to Ni (JCPDS data no. 65-380). After the process of hydriding combustion synthesis, the Mg–Ni/HCS composite mainly corresponded to MgH_2_, Mg_2_NiH_4_, and an amount of Mg that has not completely reacted. The non-appearance of Ni peaks indicated that the Ni particles reacted with Mg to form a binary intermetallic compound (Mg_2_Ni) during this process. With regard to the Mg–HCS composite, the main phases were MgH_2_ and unreacted Mg. With regard to both Mg–Ni/HCS and Mg–HCS, the non-appearance of HCS may be due to the amorphous structure of HCS and low X-ray scattering cross-sections of the light component elements.

**Fig. 3:**
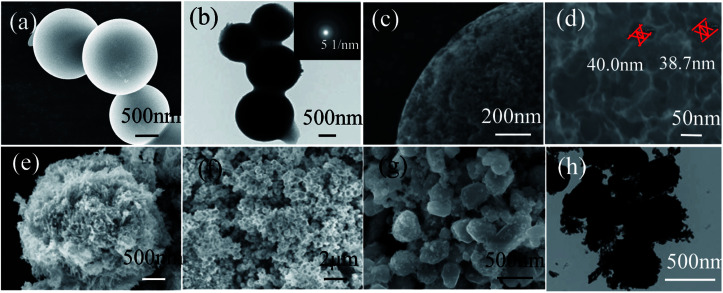
SEM images (a), (c) and (d) and TEM image (b) of the as-prepared HCS. The inset of (b) shows the corresponding selected area electron diffraction (SAED) pattern. SEM images (e) and (f) of NiO/HCS and SEM (g) and TEM images (h) of Ni/HCS. The inset of (g) shows the SEM images of Ni/HCS at different magnifications.

To obtain additional information on the valence states of Ni and HCS in Ni/HCS particles, X-ray photoelectron spectroscopy (XPS) was used (Fig. 2b–d). From the full spectrum (Fig. 2b), it is evident that the peaks of Ni were dominant; the peak observed at 854 eV corresponded to the bond of Ni 2p (Fig. 2c), demonstrating the existence of Ni.^48^ In addition, the bonding energy of 285 eV is generally assigned to the characteristic peak of C 1s (Fig. 2d).

To explore the microstructure of the products, SEM and TEM characterizations of HCS and Ni/HCS are further applied. The SEM images shown in Fig. 3a, c and d show micrographs that clearly reveal the spherical characteristics of HCS after the second carbonization process, and the majority of the particles are ±1.7 μm in diameter; some particles are fused together to form a pearl necklace, as shown in Fig. 3b. In addition, from the corresponding selected area electron diffraction (SAED) pattern (inset of Fig. 3b), we can see a dispersion center, suggesting the amorphous nature of HCS and confirming the XRD result (Fig. 1a). The surface of HCS is not smooth, and many pores (*ca.* 40 nm) can be observed on the surface of the spherules (Fig. 3c). The surface roughness increases the specific surface area of HCS, which facilitates the adsorption of Mg and Ni. This is further utilized to fabricate MgH_2_ particles or Ni/HCS. SEM observations show wire-like clusters of NiO with an average length of 500 nm and width of 50 nm, and these are uniformly anchored on the surface of HCS (Fig. 3e and f). In the solvent, HCS are assumed to act as nucleation sites, and the particles coalesce and grow, leading to the formation of NiO in the next step. However, after the reduction procedure in an H_2_ atmosphere, the wire-like clusters of NiO are converted to hexagonal Ni particles with size of around 400–500 nm (Fig. 2g and h), and HCS burst into small particles covered on the surface of Ni, reducing the agglomeration of Ni particles during the dehydrogenation process.

**Fig. 4:**
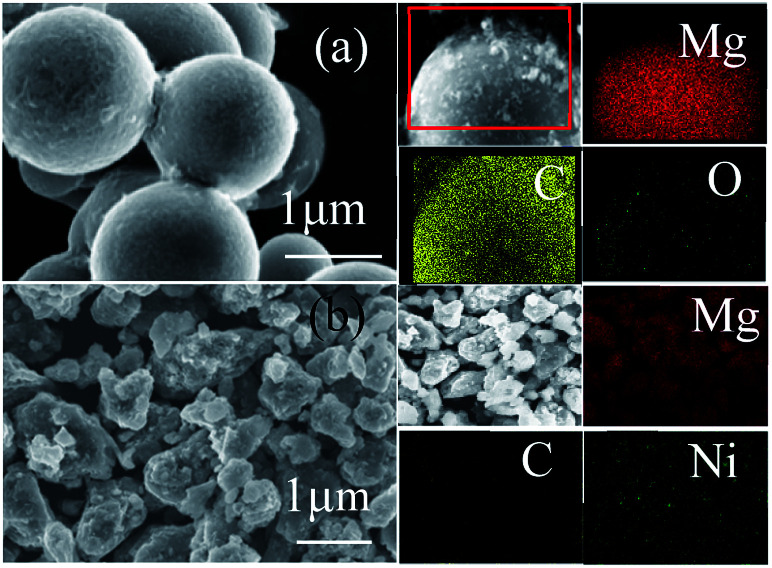
SEM images of Mg–HCS (a) and Mg–Ni/HCS (b) before ball milling. Image of the composites captured by a gallium ion beam, and the corresponding elemental mapping images of Mg, C, O, and Ni in the selected area.

SEM examinations were used to characterize the structure and distribution of the products. Interestingly, the element mapping images of the Mg–Ni/HCS composite, as shown in Fig. 4b, demonstrated that the distribution of C, Mg, and Ni was uniform without any detectable aggregation, proving that the distribution of C, Mg, and Ni composite could exhibit the entire distribution in the Mg–Ni/HCS matrix. From Fig. 4a, it can also be seen that Mg–HCS exhibited extremely fine particles on the surface of HCS, and the homogeneous anchoring was due to the favorable absorption of MgH_2_ particles on HCS. Furthermore, no aggregation was observed due to high surface area of HCS, which also acted as the structural support. Furthermore, the elemental distributions of the hydrogenated products confirmed the existence of Mg, C, and O. The results suggested that the formed MgH_2_ was well distributed and anchored on HCS owing to the favorable adsorption of Mg on HCS, which was easily realized by treatment with hydrogen. Therefore, the above analysis strongly suggested that MgH_2_ has been successfully incorporated onto the surface of HCS.

**Fig. 5:**
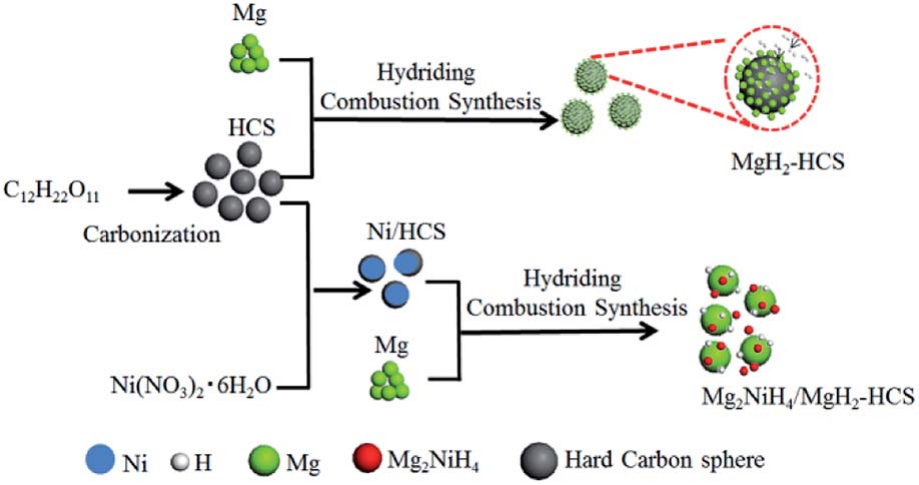
Schematic diagram of the preparation of Mg–HCS and Mg–Ni/HCS.

One of the biggest challenges is the fabrication of Mg-based hydride composites that are highly reactive and well dispersed. Here, we use Mg as the original material and maintain the growth of Mg-based hydrides by hydriding combustion synthesis. Fig. 5 shows a schematic illustration of the synthesis processes for Mg–Ni/HCS and Mg–HCS. For the Mg–Ni/HCS sample, we select sucrose as the precursor and obtain HCS *via* the carbonation process. The Ni/HCS additive is synthesized through a chemical method. During the hydriding combustion synthesis process, Mg_2_NiH_4_ particles are formed *in situ* and homogenously distributed onto the Mg/MgH_2_ matrix with the existence of HCS. In the case of Mg–HCS, Mg powder and HCS are used as the starting materials to fabricate Mg–HCS. HCS with defective domains and mesoporous characteristics favors the nucleation and growth of MgH_2_ particles. After the hydriding combustion synthesis process, MgH_2_ particles are believed to be generated on the surface of HCS, and it can easily capture hydrogen molecules and dissociate H_2_ to atomic H in the hydrogenation process after ball milling.

### 3.2 Hydrogen storage properties of Mg–Ni/HCS and Mg–HCS

**Fig. 6:**
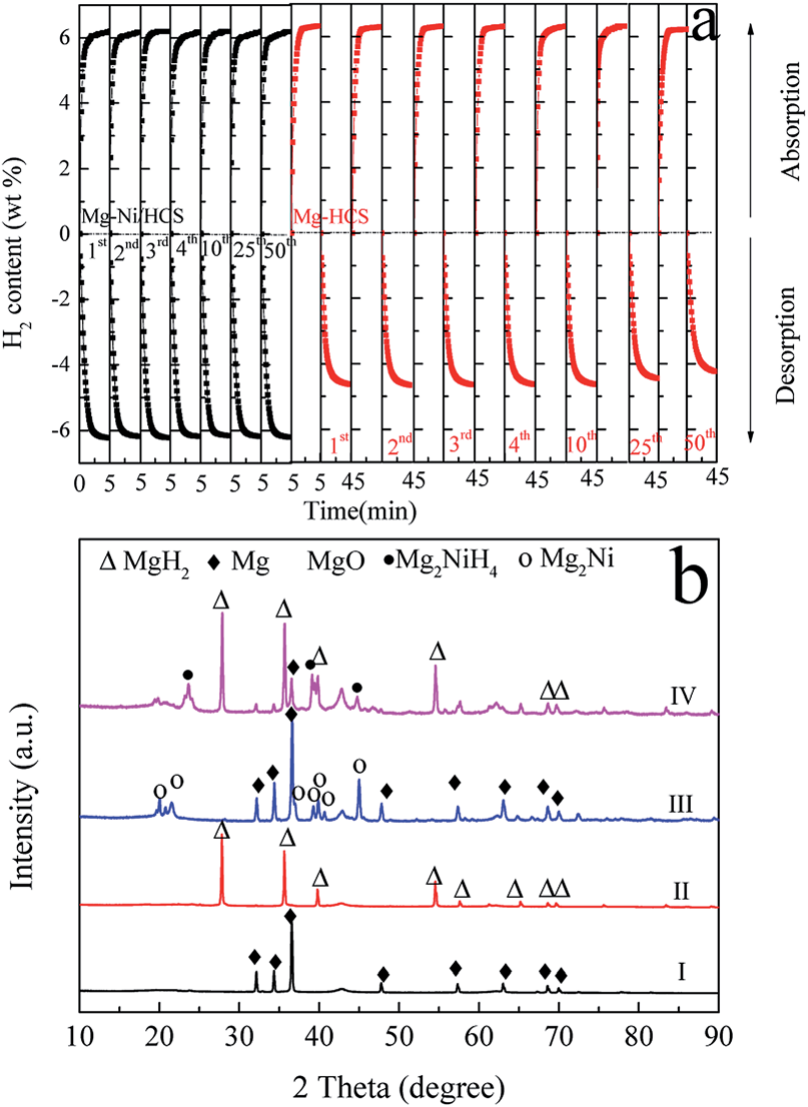
Cyclic kinetic measurements (a) of Mg–HCS and Mg–Ni/HCS composites. XRD patterns (b) of Mg–HCS (I) after dehydrogenation and (II) after rehydrogenation and those of Mg–Ni/HCS (III) after dehydrogenation and (IV) after rehydrogenation at 350 °C.

To investigate the hydrogen storage properties of the obtained Mg–Ni/HCS and Mg–HCS composites, the cyclic stability and dehydrogenation kinetic measurements are performed using PCT. First, a total of 50 cycles are performed, and the results are shown in Fig. 6a. This shows that there is no clear reduction in the capacity after 50 cycles of both Mg–Ni/HCS and Mg–HCS composites, indicating that the composites have excellent cyclic stabilities. The Mg–Ni/HCS and Mg–HCS systems are able to absorb 6.2 wt% of hydrogen within 5 min during the de/rehydrogenation process, exhibiting favourable hydrogen-absorbing dynamic performance. Meanwhile, for the MgH_2_–HCS composite, the cycling hydrogen desorption capacity is clearly lower than the absorption capacity, mainly because of the barrier induced by the breakage of the Mg–H bonds during the dehydrogenation process; therefore, for the cyclic stability measurements, between dehydrogenation and hydrogenation of each cycle, a 30 min evacuation process is conducted to ensure complete dehydrogenation reaction of the sample. Fig. 6b shows the XRD patterns of Mg–HCS and Mg–Ni/HCS composites before and after dehydrogenation after cycling. As shown in Fig. 6b (I) and Fig. 6b (III), the two samples mainly contain Mg phase after dehydrogenation, and the Mg_2_Ni phase is detected for the Mg–Ni/HCS composite, which reveals that the addition of Ni changes the path of dehydrogenation. The XRD patterns of the cycled Mg–HCS and Mg–Ni/HCS composites after hydrogen absorption are shown in Fig. 6b (II) and Fig. 6b (IV). The diffraction intensity of the MgH_2_ phase is detected, and the Mg_2_NiH_4_ phase is detected for the Mg–Ni/HCS composite. More importantly, the diffraction peaks of Mg can be detected in Fig. 6b (IV), which reveals incomplete hydrogenation of Mg. This also proves the reduction of hydrogen storage capacity during hydrogen desorption/absorption cycling. In contrast, no Mg diffraction peaks are observed for the Mg–HCS composite after hydrogenation, implying complete hydrogenation of Mg. Mao *et al.*^49^ investigated the hydrogen sorption properties of MgH_2_ doped with NiCl_2_ and CoCl_2_; they found that the MgH_2_/NiCl_2_ sample showed better sorption properties than the CoCl_2_-doped sample. As reported by Zhang *et al.*,^50^ it is experimentally confirmed that the MgH_2_–Ni_3_C composites exhibit the best hydrogen storage properties as compared to MgH_2_–Ni_3_N, MgH_2_–NiO, and MgH_2_–Ni_2_P. They suspected that Ni binding with an element having low electronegativity is favorable for the dehydrogenation of Mg-based composites, which is consistent with our finding. Moreover, Sulaiman *et al.*^51^ showed that 5 wt% of K_2_NiF_6_ had a favourable catalytic effect in the context of MgH_2_ hydrogen storage properties, which can be due to the *in situ*-formed KF, KH, and Mg_2_Ni; these active species can provide a synergetic catalytic effect in enhancing the hydrogen sorption properties of MgH_2_. Furthermore, Yahya *et al.*^52^ studied the synergistic catalytic effect of SrTiO_3_ and Ni on the improvement of the hydrogen storage properties of the MgH_2_ system; the composite can absorb 2.9 wt% of hydrogen in 10 min under pressure of 2.7 MPa of hydrogen.

It is noteworthy that with regard to the as-prepared composites, there are no indications of the formation of any metastable phases under the current experimental conditions based on XRD analysis (Fig. 6b). Furthermore, the XRD results show that no reaction occurs between Mg-based hydrides and HCS. Therefore, it is reasonable to assume that HCS with a mesoporous and defective structure may function as a dispersant, serving as the center for nucleation and growth of the magnesium hydride phase. However, a broad diffraction peak at around 42.9° can be assigned to MgO, which is consistent with the SAED results (Fig. 5). House *et al.* found that during the ball milling process, an ultrafine-grained MgO layer can be formed.^53^ Alternatively, the appearance of MgO may be ascribed to exposure to air during the measurement. It is also found that MgH_2_ is covered with oxides during desorption, demonstrating that MgO can recombine atomic hydrogen. Therefore, the existence of MgO can provide small surface vacancies, which are countered by a very high turnover frequency due to small activation energy for dissociation of hydrogen.^54^

**Fig. 7:**
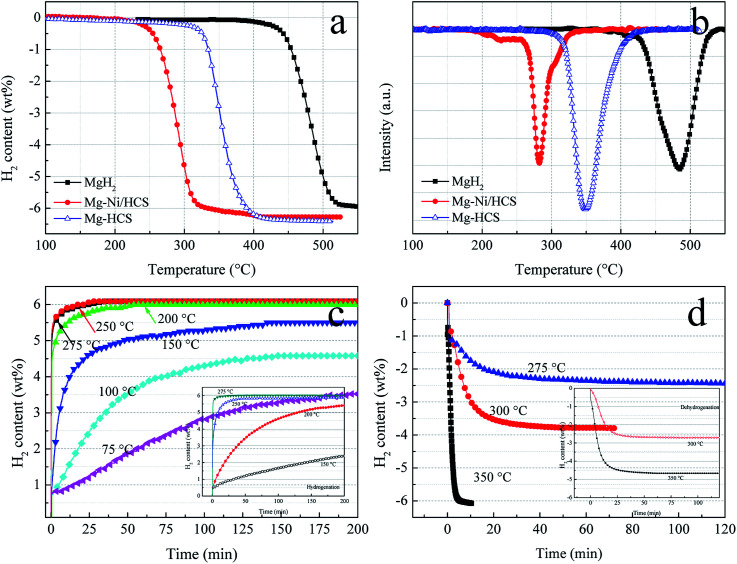
TPD curves (a) and (b) of pure MgH_2_, Mg–HCS, and Mg–Ni/HCS composites. Hydrogenation (c) and dehydrogenation (d) curves of Mg–Ni/HCS composite at various temperatures. The insets of (c) and (d) show the hydrogenation and dehydrogenation curves of Mg–HCS composite at various temperatures, respectively.

To investigate the hydrogen storage properties of Mg–Ni/HCS and Mg–HCS composites, dehydrogenation and hydrogenation measurements under different conditions are obtained using PCT. TPD experiments can provide a clear comparison of the desorption kinetics; thus, we first obtain TPD measurements to study the kinetics and overall hydrogen storage behavior. Fig. 7a shows the TPD profiles of the as-prepared Mg–Ni/HCS and Mg–HCS and milled as-received commercial MgH_2_. The dehydrogenation of Mg–Ni/HCS composite starts at about 230 °C, which is much lower than those of Mg–HCS (320 °C) and commercial MgH_2_ (425 °C). In particular, both Mg–Ni/HCS and Mg–HCS composites show a drastically faster dehydrogenation rate than primary MgH_2_. As illustrated in Fig. 7a and b, a total mass loss of 5.9 wt% is observed for the primary MgH_2_ composite with a desorption peak at 484 °C. However, the desorption peaks of Mg–Ni/HCS and Mg–HCS shift downward to 290 °C and 348 °C, respectively; both of these materials exhibit H_2_ capacity of 6.2 wt%, which is fairly close to the theoretical value of the composite (7.6 wt% × 5/6 = 6.33 wt%). This suggests that the Mg phase can completely transform into the MgH_2_ or Mg_2_NiH_4_ phase during the hydrogenation process, which agrees with the XRD results (Fig. 6b). The isothermal hydrogenation curves of the Mg–Ni/HCS composite (Fig. 7c) reveal that the hydrogenation process of the composite can be implemented within 30 min at 250 °C, and there can be 3.5 wt% hydrogen uptake even at 75 °C within 200 min. In the case of dehydrogenation plots (Fig. 7d), the temperatures increase from 275 to 350 °C; the dehydrogenation rates and capacities increase remarkably, and the excellent absorption rate remains almost unchanged. For instance, the composite can release 3.8 wt% H_2_ at 300 °C within 30 min. The hydrogen desorption behaviors of the Mg–HCS composite are also given (inset in Fig. 7(c) and (d)). For hydrogen absorption, the Mg–HCS composite can reach the maximum value in less than 5 min; for example, it can uptake 2.5 wt% H_2_ at 150 °C. Although this value is enhanced when compared with that of pure MgH_2_, it is much lower than that for the Mg–Ni/HCS composite. Therefore, the absorption properties are clearly improved by introducing Ni/HCS, suggesting the prominent effect on the hydrogenation reaction of Mg. By further increasing the applied temperature, the desorbed hydrogen capacity significantly increases (Fig. 7d), indicating that the performance of dehydrogenation is considerably influenced by the temperatures of both Mg–Ni/HCS and Mg–HCS composites.

**Fig. 8:**
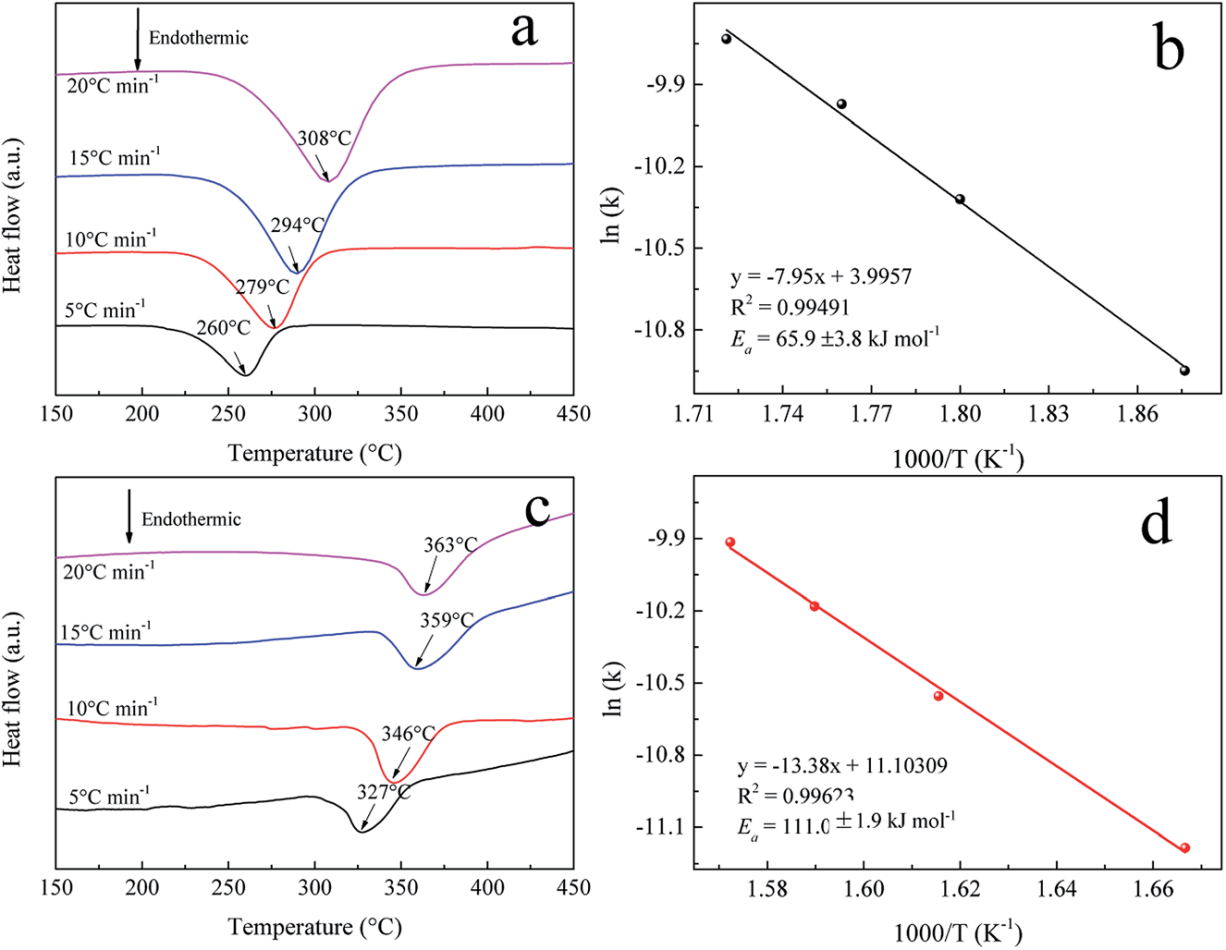
DTA profiles at different heating rates of (a) Mg–Ni/HCS and (c) Mg–HCS. Kissinger plots of the dehydrogenation of (b) Mg–Ni/HCS and (d) Mg–HCS.

To further understand the hydrogenation kinetics mechanism, we also analyzed the DTA profiles at different ramping rates and estimated the activation energies (*E*_a_) of the dehydrogenation processes of Mg–Ni/HCS and Mg–HCS based on the Kissinger method according to eqn (1). Here, *α* is the heating rate, *T*_m_ is the peak temperature, and *R* is the gas constant. In addition, *T*_m_ was obtained at *α* values of 5, 10, 15, and 20 °C min^−1^, as shown in Fig. 8a and c. Fig. 8b and d show the slopes of the fitted lines of Mg–Ni/HCS and Mg–HCS, respectively, which are used to determine the value of *E*_a_/*R*. The *E*_a_ value for the Mg–Ni/HCS composite was 65.9 kJ mol^−1^; the *E*_a_ value for the Mg–HCS sample was 111.0 kJ mol^−1^ and that for pure MgH_2_ was 133.5 kJ mol^−1^.^55^ The values for the Mg–Ni/HCS and Mg–HCS composites were 67.6 kJ mol^−1^ and 22.5 kJ mol^−1^ lower than that for pure MgH_2_, respectively, which provided further evidence for the correlation between the presence of HCS and hydrogen desorption kinetics as well as the synergistic catalysis between HCS and Ni.1
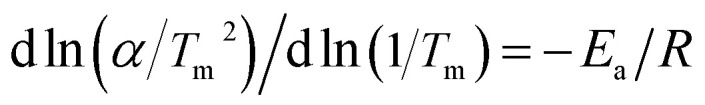


**Fig. 9:**
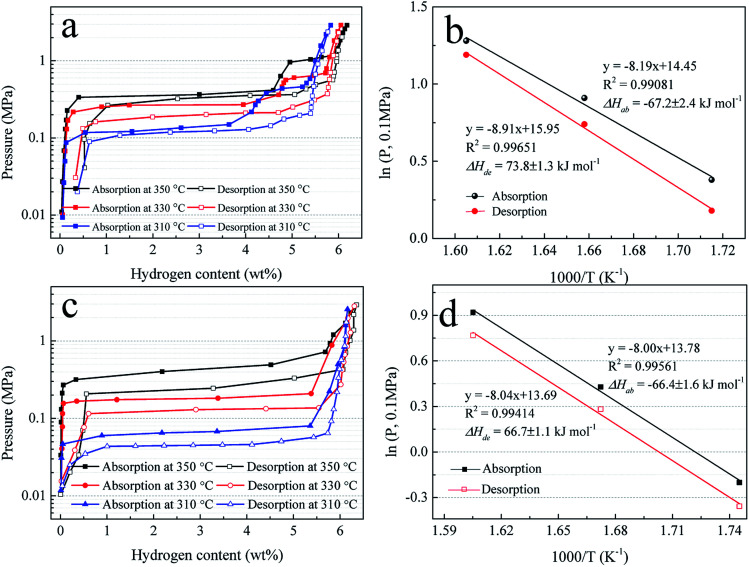
*P*–*C* isotherms for hydrogen absorption and desorption (a) and (c) and Van't Hoff plots, *i.e.*, log *P*(H_2_) *vs.* 1000/*T* (b) and (d) of Mg–Ni/HCS and Mg–HCS.

To further study the hydrogen absorption and desorption thermodynamics, the pressure–composition (*P*–*C*) isotherms of Mg–Ni/HCS and Mg–HCS are measured at 350, 330, and 310 °C, as shown in Fig. 9a and c, respectively. The Van't Hoff plots derived from the *P*–*C* isotherms are shown in Fig. 9b and d. For the Mg–Ni/HCS composite, two plateaus are clearly visible for each plot, independent of the temperature; they correspond to the hydrogen sorption reactions for Mg/MgH_2_ at a lower concentration of H_2_ (long and fairly flat plateau) and hydrogen sorption reactions for Mg_2_Ni/Mg_2_NiH_4_ (short and narrow plateau) at a higher concentration of H_2_. Based on the midpoint pressures at different temperatures, Mg–Ni/HCS displays the equilibrium pressures of absorption plateaus at lower plateaus of 1.46, 2.48, and 3.60 bar, and the equilibrium pressures of the desorption plateaus are 1.23, 2.12, and 3.30 bar at 310, 330, and 350 °C, respectively. The hydrogenation and dehydrogenation enthalpies (Δ*H*) are determined to be −67.2 and 73.8 kJ mol^−1^ H_2_, and the hydrogenation and dehydrogenation entropies are calculated to be −119.5 and 131.9 J K^−1^ mol^−1^ H_2_, respectively. As compared to the standard value for MgH_2_ (74.7 kJ mol^−1^ H_2_),^56^ the Δ*H* values for hydrogenation and dehydrogenation are not essential. For the Mg–HCS composite, only one plateau is identified at each temperature, corresponding to the reactions for the Mg/MgH_2_ system. Based on a similar method, the hydrogenation and dehydrogenation enthalpies of the Mg–HCS composite are determined to be about −66.4 and 66.7 kJ mol^−1^ H_2_, and the hydrogenation and dehydrogenation entropies are determined to be about −114.3 and 113.6 J K^−1^ mol^−1^ H_2_, respectively. When compared to the standard value for MgH_2_ (74.7 kJ mol^−1^ H_2_), the Δ*H* values for hydrogenation and dehydrogenation are also not prominent, suggesting marginal changes in the thermodynamic properties following the addition of HCS and Ni/HCS.

### 3.3 Mechanism

The above performances of Mg–Ni/HCS and Mg–HCS composites prepared by hydriding combustion synthesis and ball milling reveal considerably enhanced hydrogen storage properties of Mg hydrides. Nevertheless, the PCT measurement results exhibited marginal differences in the thermodynamic properties. We highlight the excellent kinetic improvements in the as-prepared Mg–Ni/HCS composite in the dispersity of HCS and the *in situ*-formed Mg_2_NiH_4_. The particular distribution of the Mg_2_NiH_4_ phase is assumed to play a critical role in triggering de/absorption of MgH_2_. Zhang *et al.*^57^ found that in Ni-containing Mg-based systems, Ni always transforms into Mg_2_NiH_4_ during the hydriding combustion synthesis process, and it changes to Mg_2_Ni after desorption; the Mg_2_Ni phase can act as a porthole to adsorb H_2_ and dissociate H_2_ into H atoms. This yields more significant reduction in the hydrogenation barrier as compared to that for pure Mg and triggers its hydrogen performances. Please note that HCS burst and wrap onto Ni; therefore, the existence of HCS causes a sufficient dispersion effect in the hybrid. The active sites of carbon materials are also crucial for facilitating the nucleation of Mg_2_Ni/Mg_2_NiH_4_ particles.

## 4. Conclusions

We have prepared an Mg–Ni/HCS composite by hydriding combustion synthesis and mechanical milling process involving Mg powder and carbon-wrapped Ni additive as the raw materials. Microstructural characterization reveals that the *in situ*-formed Mg_2_NiH_4_ particles are evenly distributed into the matrix of the composite and play a key role in significant enhancement of hydrogen desorption kinetics. HCS assume an important role as a support material in providing nucleation sites for MgH_2_ and Mg_2_NiH_4_ and act as an inhibitor for the agglomeration of hydrides to enhance the dehydrogenation performances. The Mg–Ni/HCS composite absorbs 3.5 wt% of hydrogen in 200 min at 75 °C and desorbs 2.7 wt% of hydrogen in 120 min at 275 °C. The *E*_a_ value of the composite for the dehydrogenation of MgH_2_ is 65.9 kJ mol^−1^, indicating sharp reduction in the barrier to dehydrogenation when compared with that of commercial MgH_2_. Our results demonstrate that the carbon-wrapped Ni additive can greatly improve the hydrogen performances of the Mg-based hydride system and provide considerable potential for practical applications.

## Conflicts of interest

There are no conflicts to declare.

## Supplementary Material
